# Combining targeted therapy and immune checkpoint inhibitors in the treatment of metastatic melanoma

**DOI:** 10.7497/j.issn.2095-3941.2014.04.002

**Published:** 2014-12

**Authors:** Teresa Kim, Rodabe N. Amaria, Christine Spencer, Alexandre Reuben, Zachary A. Cooper, Jennifer A. Wargo

**Affiliations:** ^1^Department of Surgery, Massachusetts General Hospital, Boston, MA 02114, USA; ^2^Harvard Medical School, Boston, MA 02115, USA; ^3^Department of Melanoma Medical Oncology, ^4^Department of Genomic Medicine, ^5^Department of Surgical Oncology, University of Texas M.D. Anderson Cancer Center, Houston, TX 77030, USA

**Keywords:** Melanoma, checkpoint blockade, BRAF inhibition, immunotherapy

## Abstract

Melanoma is the deadliest form of skin cancer and has an incidence that is rising faster than any other solid tumor. Metastatic melanoma treatment has considerably progressed in the past five years with the introduction of targeted therapy (BRAF and MEK inhibitors) and immune checkpoint blockade (anti-CTLA4, anti-PD-1, and anti-PD-L1). However, each treatment modality has limitations. Treatment with targeted therapy has been associated with a high response rate, but with short-term responses. Conversely, treatment with immune checkpoint blockade has a lower response rate, but with long-term responses. Targeted therapy affects antitumor immunity, and synergy may exist when targeted therapy is combined with immunotherapy. This article presents a brief review of the rationale and evidence for the potential synergy between targeted therapy and immune checkpoint blockade. Challenges and directions for future studies are also proposed.

## Introduction

### Epidemiology of melanoma

Skin cancer is among the most common cancer types globally^1^. Melanoma is the deadliest skin cancer^2^. Its burden on public health continues to rise, with its incidence increasing faster than any other cancer in recent years^1^^,^^2^. Early stage melanoma is treatable with surgery, but the late stage of this disease is often fatal^3^. This review discusses the treatment options for patients with advanced melanoma and the rationale for combining targeted therapy and immune checkpoint inhibitors to treat these patients.

### Treatments for advanced melanoma

#### Chemotherapy

Treatment options for advanced melanoma were limited in the past decade, and prognosis was universally poor. Cytotoxic chemotherapy was the main treatment strategy but was marginally effective only in the treatment of locally advanced or metastatic disease. Dacarbazine [5(3,3-dimethyl-1-triazeno)-imidazole-4-carboxamide] was the primary agent used, and this drug remains the only FDA-approved chemotherapy for metastatic melanoma. However, therapy is characterized with low overall response rates (approximately 10%-15%), and the drug offers no survival benefit^4^^,^^5^.

In addition to dacarbazine treatment, biochemotherapy regimens and combined chemotherapeutic agents (e.g., dacarbazine or temozolomide with vinblastine and cisplatin) with cytokine-based therapy [e.g., interleukin-2 (IL-2) and interferon-alpha] had been administered to improve response rates by inducing immunogenic cell death. Two large meta-analyses that evaluated the standard chemotherapy versus biochemotherapy in 18 randomized controlled trials (RCTs) involving more than 2,600 patients with metastatic melanoma show that biochemotherapy regimens can improve overall response rates, but with greater systemic toxicity and without a statistically significant survival benefit^6^^,^^7^.

#### Immunotherapy

Another modality in melanoma treatment involves the use of immunotherapy. The first immune-based therapy with demonstrated clinical benefit in melanoma patients was IL-2, an immune stimulating cytokine integral to T cell activation and proliferation. Atkins *et al*.^8^ examined 270 patients with metastatic melanoma treated with high dose IL-2, and 16% of patients who achieved complete response (CR) or partial response (PR) showed long-term responses, with a median progression free survival (PFS) of 13.1 months. Longer follow-up time of the patients demonstrated an approximately 6% CR rate^9^. A follow-up phase III RCT by Schwartzentruber *et al*.^10^ demonstrated a small but statistically significant improvement in objective response rate (ORR) associated with the addition of the gp100 peptide vaccine to high dose IL-2, although, again, in only a small percentage of patients (16% *vs*. 6%) treated with vaccine plus IL-2 versus IL-2 alone.

Immune checkpoint inhibitors have also been successfully used to treat melanoma. This therapy is based on the fact that T lymphocytes are critical to antitumor immunity, and activation by an antigen-specific T cell receptor in the context of costimulatory activation is required. However, a naturally occurring feedback mechanism that prevents excess immune activation through the expression of negative costimulatory molecules exists^11^. These negative costimulatory molecules, also known as “checkpoints”, such as cytotoxic T-lymphocyte antigen 4 (CTLA-4), programmed death 1 (PD-1), T cell immunoglobulin 3, and lymphocyte-activation gene 3, act as “brakes” on T cell activation and serve as negative feedback mechanism^11^. Interestingly, tumor-infiltrating T lymphocytes (TIL) in many tumor types express high levels of negative costimulatory markers, suggesting a tumor-derived mechanism of suppressing antitumor immunity and providing rationale for T cell checkpoint blockade^12^.

In a milestone phase III RCT of 676 patients with unresectable stage III or IV melanoma treated with either anti-CTLA-4 antibody ipilimumab, gp100 peptide vaccine, or combined ipilimumab plus vaccine, patients treated with either ipilimumab arm had improved overall survival (OS) compared with those treated with vaccine alone (10.0 *vs*. 6.4 months)^13^. Ipilimumab alone achieved the best overall response rate in 10.9% of patients, and 60% of these patients benefitted from long-term responses lasting greater than 2 years. However, ipilimumab therapy was also associated with higher toxicity rate, with 10%-15% of patients suffering from grade 3 or 4 immune-related adverse events (AEs) such as diarrhea or colitis, dermatitis, and pruritis^13^. Similar results were reported in a second RCT, which compared ipilimumab plus dacarbazine versus dacarbazine alone in 502 patients with advanced melanoma, but this study utilized a higher dose (10 mg/kg) of ipilimumab^14^. Response rates were 15% in the ipilimumab with dacarbazine-treated group but with higher toxicities. Grade 3 or 4 AEs occurred in 56% of patients^14^.

Topalian *et al*.^15^ recently reported the results of the phase I trial of 296 patients with either advanced melanoma or other solid tumors, which included non-small cell lung cancer, prostate cancer, renal cell carcinoma, and colorectal cancer, in which the checkpoint blocking antibody anti-PD-1 (BMS-936558, nivolumab) achieved a 28% response rate in melanoma patients, with long-term responses longer than 1 year in 50% of responding patients. Anti-PD-1 therapy was associated with a lower rate of grade 3 or 4 AEs compared with ipilimumab. Interestingly, Topalian *et al*.^15^ suggested a possible association between tumor expression of the PD-1 ligand PD-L1 and response to anti-PD-1 therapy. However, further studies are necessary to confirm this finding. In a pooled analysis of 411 melanoma patients treated with the anti-PD-1 antibody MK-3475 (pembrolizumab, Merck Sharpe & Dohme) with over 6 months of follow-up data, the ORR was 40% in ipilimumab naïve patients and 28% in ipilimumab refractory patients^16^. Median PFS was 24 weeks, but median OS had not been reached at the time of analysis. Pembrolizumab was well-tolerated with 12% of patients experiencing a grade 3 or 4 AE attributed to the drug, but only 4% of patients discontinued treatment because of AE^16^. This agent was FDA-approved for metastatic melanoma in early September 2014. The antibody blockade of PD-L1 in the phase I trial of 207 patients using BMS-936559, including 55 with advanced melanoma, achieved an objective response in 17% of melanoma patients, with more than half of patients achieving long-term responses lasting longer than 1 year and a comparable rate of grade 3 or 4 AEs^17^. Although this agent is currently not being tested, two anti-PD-L1 antibodies, namely, MPDL3280A (Genentech) and MEDI4736 (MedImmune), are being tested in solid tumors under early phase clinical trials.

In addition to their use for monotherapy, different immune checkpoint inhibitors are now being combined in clinical trials, which showed impressive response rates. In the phase I trial of 86 patients with unresectable stage III or IV melanoma treated with either concurrent or sequential ipilimumab and nivolumab, concurrent CTLA-4 and PD-1 blockade achieved a higher ORR of 40%, with 53% of patients achieving CR or PR at the maximum doses tested, whereas 31% of responders demonstrated tumor regression of 80% or more even with bulky disease^18^. In 53% of patients, grade 3 or 4 AEs occurred at a higher frequency in combined therapy than in monotherapy, the majority of which were reversible with appropriate supportive management^18^. In a recent update, the 1-year OS rate for patients treated with combined immune checkpoint inhibitors was 85%, and their 2-year survival rate was 79%^19^.

Additional strategies targeting checkpoint inhibitors and other immunomodulatory molecules are currently being studied. However, a thorough discussion of these strategies (as well as other strategies such as treatment with tumor-infiltrating lymphocytes) is beyond the scope of this review.

#### Targeted therapy

Concurrent advances in targeted molecular therapy have also improved the treatment and prognosis of a subset of advanced melanoma patients. Approximately 50% of cutaneous melanomas harbor an activating mutation in the *BRAF* oncogene, leading to constitutive activation of the mitogen-activated protein kinase (MAPK) signaling pathway involved in cellular proliferation and survival^20^. Preclinical studies of vemurafenib (PLX4032), a potent oral small molecule inhibitor of mutated BRAF, have culminated in a phase III RCT of vemurafenib versus dacarbazine in 675 patients with *BRAF*-mutated metastatic melanoma^21^. The vemurafenib arm resulted in improved OS (84% *vs*. 64% at 6 months) and higher response rate (48% *vs*. 5%) than standard chemotherapy, representing the only treatment other than anti-CTLA-4 to improve survival in metastatic melanoma^21^. Similar results were demonstrated in a phase III RCT, in which another potent BRAF^V600E^ inhibitor, dabrafenib, was compared with dacarbazine^22^. Despite improvements in response rate and survival, BRAF inhibition achieved only a median PFS of 6 months, implying rapid development of tumor resistance^21^^,^^22^. Further investigation of resistance mechanisms has suggested that *BRAF*-mutated melanoma cells can maintain MAPK signaling through RAF-independent activation of MEK, a kinase downstream of RAF in the MAPK cascade^23^. Translating these findings to clinical studies, Flaherty *et al*.^24^ demonstrated that combined BRAF and MEK inhibition (dabrafenib plus trametinib *vs*. dabrafenib alone)achieved a higher overall response rate of 76% versus 54%, as well as a longer median PFS of 9.4 *vs*. 5.8 months. Similar to immunotherapy, combination molecular therapy, which targets multiple levels of an oncogenic signaling cascade or multiple different cell survival pathways, will likely enhance tumor response. In fact, dual immune and molecular therapy together may offer the best possibility of both long-term and frequent response^25^.

#### Rationale for combination therapy

Newly approved targeted and immune-modulating agents have provided numerous treatment options. However, the optimal sequencing of these agents remains controversial. Even though BRAF inhibition through selective BRAF inhibitors produces excellent early disease control for patients with V600E/K mutations, the response duration of this approach is limited to less than a year because of the development of multiple molecular mechanisms of resistance^23^^,^^26^^-^^32^. Checkpoint blockade with the CTLA4 inhibitor, ipilimumab, and anti-PD-1 antibodies produces responses in a minority of patients, but with long-term responses^13^^,^^15^. Thus, the combination of targeted therapy and immunotherapy may lead to early and robust antitumor responses from targeted therapy with long-term benefit from the influence of immunotherapy.

## Preclinical data

### *In vitro* studies

To date, numerous studies have investigated combined targeted therapy and immunotherapy in melanoma. The first report suggesting that oncogenic BRAF^V600E^ can lead to tumoral immune escape was published in 2006^33^. Further *in vitro* studies have been performed after the development of specific BRAF inhibitors, and BRAF inhibition in BRAF mutant melanoma cell lines and fresh tumor digests has been demonstrated to result in up regulation (up to 100-fold) of melanoma differentiation antigens^34^. Additionally, inhibition with BRAF and MEK inhibitors increased the recognition of these melanoma antigens by antigen-specific T lymphocytes. However, MEK inhibitors adversely affect the T cell function whereas those treated with BRAF inhibitors maintained functionality^34^. Further independent studies on the effects of dabrafenib (BRAF inhibitor), trametinib (MEK inhibitor), or their combination on T lymphocytes have also shown that trametinib alone or in combination suppressed T-lymphocyte proliferation, cytokine production, and antigen-specific expansion, whereas treatment with dabrafenib had no effect^35^. Callahan *et al*.^36^ suggested that BRAF inhibitors can modulate T cell function by potentiating T cell activation through ERK signaling *in vitro* and *in vivo*.

### *In vivo* studies

Importantly, the effect of BRAF inhibition has also been studied in patients with metastatic melanoma. Results showed a similar increase in melanoma differentiation antigens and a significant increase in intratumoral CD8^+^ T cells, which were more clonal 10-14 days after initiation of BRAF inhibition^37^^-^^39^. These findings were also associated with down regulated IL-6, IL-8, IL-1α and vascular endothelial growth factor (VEGF)^38^^,^^40^^,^^41^. The increased immunomodulatory molecules, PD-1 and PD-L1, 10-14 days after BRAF inhibition initiation are also important, and this condition suggests a potential immune-based mechanism of resistance^38^. The up regulated PD-L1 expression may have been caused by infiltrating IFN-γ-secreting T cells^42^, although stromal components may also be involved^43^. Jiang *et al*.^44^ also suggested PD-L1 expression as a mechanism of resistance to BRAF inhibitors because BRAF-resistant cell lines expressed high PD-L1, and the addition of MEK inhibitors has a suppressive effect on PD-L1 expression. These data indicate that addition of immunotherapy and specifically immune checkpoint blockade may enhance antitumoral response when combined with a BRAF inhibitor.

Mouse models have provided important insights into cancer development, treatment, and therapeutic resistance. Several preclinical mouse models have been used to examine in detail the potential of combining immunotherapy with BRAF-targeted therapy, and most studies have indicated an additive or synergistic effect. In the syngeneic SM1 mouse model of BRAF^V600E^ melanoma, an improved antitumor activity was observed after combining BRAF inhibition with adoptively transferred T cells, leading to increased *in vivo* cytotoxic activity and intratumoral cytokine secretion by the transferred T cells. Interestingly, BRAF inhibition did not alter adoptively transferred T cell expansion, distribution, or intratumoral density^45^. Liu *et al*.^41^ also studied the effects of BRAF inhibition on adoptively transferred cells by using pmel-1 TCR transgenic mice on a C57BL/6 background and xenografts of melanoma cells transduced with gp100 and H-2Db. They found an increase in T cell infiltration, which was associated with VEGF. Additionally, they showed that melanoma cell VEGF over expression abrogated T cell infiltration, and these findings were validated in patients treated with BRAF-directed therapy considering that down regulated intratumoral VEGF is correlated with a denser intratumoral T cell infiltrate after melanoma patients were treated with BRAF inhibitors^41^. Knight *et al*.^46^ utilized several mouse models, which included SM1, SM1WT, and a transgenic mouse model of melanoma, to support the therapeutic potential of combining BRAF inhibitors and immunotherapy. They observed an increase in CD8:Treg ratio after BRAF inhibition and that depleting CD8^+^ T cells, not NK cells, was required for antitumor activity of BRAF inhibitors. They also showed that CCR2 demonstrates an antitumoral role after BRAF inhibition and that combination of BRAF-targeted therapy and anti-CCL2 or anti-CD137 led to a significant increase in antitumoral activity in these mouse models^46^.

The authors recently demonstrated a potential synergy when immune checkpoint blockade was added to BRAF inhibition^47^. This process was performed using a BRAF^V600E^/PTEN^–/–^syngeneic subcutaneous mouse model, which showed an increase in intratumoral CD8 T cells after BRAF inhibitor initiation, similar to melanoma patients. Either PD-1 or PD-L1 blockade addition to BRAF inhibition resulted in enhanced response, which slowed tumor growth and enhanced survival. Additionally, increased number and activity of infiltrating TIL was observed^47^.

However, a previous study has shown the absence of synergy in combined BRAF-targeted and immunotherapy^48^. This study conducted experiments using a conditional melanocyte-specific expressed BRAF^V600E^ and PTEN gene that led to 100% penetrance, short latency, and lymph node and lung metastases. These induced tumors were similar to human melanoma tumors from a histologic standpoint, but the immune response to BRAF inhibition was distinct from that observed in BRAF-inhibitor-treated patients with metastatic melanoma^38^^,^^39^^,^^48^. In this model, treatment with anti-CTLA4 blockade and BRAF inhibition was not associated with improved survival or decreased tumor outgrowth^48^. These results are contrary to those observed in several other models. Thus, understanding the translational relevance of individual models and their utility in guiding the development of human clinical trials is important.

**Figure 1:**
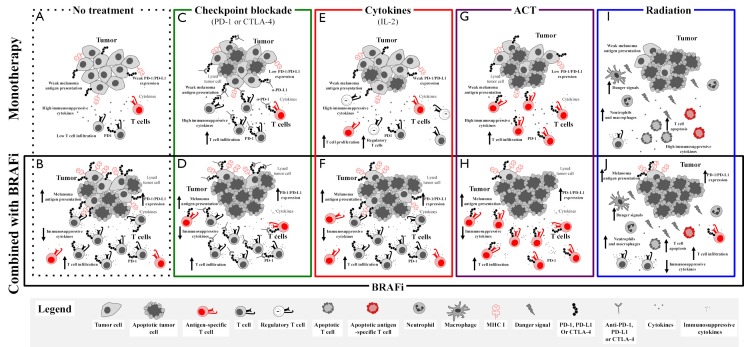
Putative effects of adding BRAF targeted therapy to immune-based therapies. Without treatment, melanomas demonstrate an immunosuppressive environment with generally low levels of melanoma antigens, low levels of infiltrating cytotoxic T lymphocytes, and high levels of immunosuppressive cytokines and VEGF (A). Treatment with a BRAF inhibitor results in a favorable tumor microenvironment with increased antigens and CD8+ T cells and decreased immunosuppressive cytokines and VEGF, but with concurrent increase in immunomodulatory molecules, such as PD-1 on T cells and PD-L1, in the tumor microenvironment (B). BRAF-targeted therapy may synergize with different treatment modalities, and this phenomenon is being tested in murine models and in clinical trials. Evidence for synergy exists with immune checkpoint blockade (C), considering that the BRAF inhibitor addition has positive effects on the tumor microenvironment (with increased antigens, CD8+ T cells, and decreased immunosuppressive cytokines/VEGF) and that the simultaneous increase in immunomodulatory molecules is tempered by immune checkpoint blockade (D). The potential synergy includes combined treatment with IL2 (E), in which the addition of BRAF-targeted therapy may augment the antitumor immune response by its favorable effects on the tumor microenvironment (F). Nevertheless, regulatory T cells in this setting may be controversial. Adoptive cell therapy works through ex vivo activation of autologous antigen-reactive T cells (G), and this behavior may be enhanced by the favorable effects of BRAF-targeted therapy on the tumor microenvironment (H). Radiation therapy has clear effects on the tumor microenvironment with effects on both tumor cells and antitumor immunity (I), which may be augmented by the addition of BRAF-targeted therapy (J).

Therefore, combined BRAF-targeted therapy with immunotherapy based on preclinical *in vitro* and *in vivo* work is advantageous. Aggregate data suggest that BRAF inhibitor treatment is associated with increased melanoma antigens, increased CD8 T cell infiltrate, and decreased immunosuppressive cytokines and VEGF early in the course of therapy (within 2 weeks of initiating treatment in patients)^38^^,^^40^^,^^41^. However, a simultaneous increase in immunomodulatory molecules was also found, which may contribute to therapy resistance. Adding BRAF-targeted therapy to a number of different treatment modalities could improve responses (**Figure 1**), and these combinations are currently being tested in murine models and clinical trials.

## Current and ongoing clinical trials of combined targeted and immunotherapy

Translating the concepts derived from previous studies has attracted much attention for application in patient care setting. However, data on how to treat patients with combined targeted and immunotherapy approaches are insufficient. A phase I study tested the combination of the BRAF inhibitor vemurafenib with the CTLA4 inhibitor ipilimumab. The first cohort of 6 patients received full dose vemurafenib at 960 mg orally twice daily for 1 month as a single agent prior to intravenous administration of ipilimumab at the FDA-approved dose of 3 mg/kg. Dose limiting toxicity (DLT) of grade 3 transaminase elevations were noted in four patients within 2-5 weeks after the first dose of ipilimumab^49^. A second cohort of patients was then started, in which patients were started on lower dose vemurafenib (720 mg orally twice daily) with full dose ipilimumab. Hepatotoxicity was again observed with grade 3 transaminase elevations in two patients and grade 2 elevation in one patient^49^. Additionally, one patient in each cohort experienced grade 2 or 3 total bilirubin elevation. All hepatic AEs were asymptomatic and reversible either with temporary discontinuation of both study drugs or with administration of glucocorticoids. Additional AEs of interest included grade 2 temporal arteritis in one patient in cohort 1 and grade 3 rash in two patients in cohort 1. The study was discontinued because of hepatotoxicity issues^49^.

An ongoing targeted and immunotherapy trial utilizes dabrafenib with or without trametinib combined with ipilimumab in patients with BRAF V600E/K-mutated metastatic melanoma (NCT01767454). At the time of data presentation at the American Society of Clinical Oncology (ASCO) meeting in June 2014, 12 patients had been enrolled on the doublet of ipilimumab with dabrafenib, and 7 patients were enrolled on triplet therapy^50^. No DLTs were found in the doublet arm of dabrafenib (150 mg) administered orally twice daily and in ipilimumab (3 mg/kg). Thus, a dose expansion of 30 additional patients is ongoing. Although hepatotoxicity was observed in the doublet arm, grade 3 or 4 toxicities were not noted, which is likely due to the lower propensity of hepatotoxicity seen with dabrafenib compared with vemurafenib^50^. Data are currently insufficient to estimate the duration of benefit from doublet therapy.

In the triplet cohort, two cases of colitis associated with colon perforation were noted in the first seven treated patients. Both patients required extensive courses of steroids, and one patient required surgery for management of the colon perforation. Toxicities were observed despite the use of low-dose dabrafenib 100 mg twice daily and trametinib 1 mg daily^50^. Accrual of patients in this cohort was suspended because of toxicity. Sequential administration of ipilimumab and trametinib in combination with dabrafenib is under consideration.

Several other studies that combine targeted therapy and immunotherapy have been planned or are underway, each with varying dose levels and schedules of combination therapy administration (**Table 1**). These important trials will aid in understanding toxicity profile and provide preliminary efficacy data of various combinations, including targeted treatment with checkpoint blockade, cytokine therapy, T cell therapy, or radiation. Many of these trials were based on the backbone of dabrafenib- and trametinib-targeted therapy with some variations on the use of BRAFi or MEKi alone. This condition is expected to establish whether MEKi is truly detrimental when combined with immunotherapy.

**Table 1:** Phase I/II studies of combining targeted and immunotherapy in melanoma

Targeted + checkpoint blockade	Targeted + cytokine	Targeted + T cells	Targeted + radiation
Dabrafenib ± trametinib + ipilimumab (NCT01767454)	Vemurafenib + high dose IL-2 (NCT01754376 and NCT 01683188)	Vemurafenib + tumor infiltrating lymphocytes (NCT00338377; NCT01585415; NCT01659151)	Dabrafenib + stereotactic radiosurgery to the brain (NCT01721603)
Vemurafenib + Anti PDL1 (MPDL3280) (NCT01656642)	Vemurafenib + IL-2 (infusional 96 hour) + INFα (NCT01603212)		Vemurafenib + whole brain radiation or radiosurgery to the brain (NCT02145910)
Dabrafenib + trametinib + anti PD1 (MK-3475) (NCT02130466)	Vemurafenib + pegylated IFN (NCT01959633)		
Trametinib ± dabrafenib + anti PDL1 (MEDI4736) (NCT02027961)	Vemurafenib + high dose IFNα-2b (NCT01943422)		

The encouraging data regarding checkpoint blockade make these agents ideal for combination with targeted therapy. Given that the side effect profiles, response rates, and durations of response differ among CTLA4, PD-1, and PD-L1 blockers, these trials will be instrumental in providing toxicity and efficacy data. Most of these trials have been designed to involve different cohorts to determine whether the combination drugs should be started simultaneously or whether targeted therapy should be administered first.

Given that cytokine therapy has long been the primary treatment for advanced stage melanoma, combined targeted treatment and cytokines are currently under clinical investigation. Combination therapy is expected to increase immune recognition of melanoma cells by CD8 T cells through up regulation of IFN-αR1 and class I HLA expression. Skin and hepatotoxicity could be overlapping for the vemurafenib and cytokine trials. Thus, efficacy and toxicity data should be ascertained.

Infusion of TIL for therapeutic benefit in patients is an active area of research interest and is among the most effective immunotherapies in melanoma with approximately 45% ORR^51^. A murine adoptive cell therapy model was utilized to illustrate that selective BRAF inhibitor PLX4720 could increase tumor infiltration of adoptively transferred T cells and enhance the antitumor activity of the T cells^41^. This process was mediated by inhibiting the production of VEGF by melanoma cells. This finding was also verified in human melanoma patient tumor samples before and during BRAF inhibition^41^. Multiple TIL with targeted therapy trials are ongoing (**Table 1**).

Selective BRAF inhibitors produce objective responses in patients with CNS disease^52^. However, data on the combined use of targeted therapy with radiation are insufficient. Although abscopal effect has been reported with use of ipilimumab and concurrent radiation^53^, this phenomenon has been less well studied with targeted therapy agents. A recent publication has reported a patient with BRAF-mutated melanoma who developed progressive disease in the brain and pelvic lymph nodes after single-agent vemurafenib treatment. Vemurafenib was discontinued, and the patient was treated with stereotactic radiosurgery (SRS) to three CNS metastases, in which imaging showed complete resolution of pelvic nodes 1 month after SRS and no evidence of CNS disease for at least 18 months. The recent use of BRAF inhibitor in this patient was assumed to have facilitated a more favorable tumor microenvironment with enhanced antigen presentation to tumor cells that was augmented with the use of SRS^54^. Together with planned immune correlative studies, these ongoing studies were designed to assess whether the addition of the BRAF-targeted agent improves disease-free survival rate compared with radiation alone to help better study the hypothesized abscopal effect.

## Future directions

Metastatic melanoma treatment has been revolutionized over the past few years with the development of immunotherapeutic and targeted agents that improve the OS of patients. Although both immunotherapy and targeted therapies have distinct advantages and disadvantages, preclinical data suggest that combinations of these treatments could further improve patient outcomes. Data of patients who were treated with combined therapy are limited. Response data are therefore insufficient to make conclusions. However, the development of toxicities has been an issue, and controversial questions remain unclear.

The optimal timing and sequence of combination therapy is currently unknown. Trials are being conducted to ascertain whether the agents should be administered simultaneously or targeted agents should be used first to prime the T cell response. Serial biopsies in a single patient on combined vemurafenib and ipilimumab showed increased T cell infiltrate and increase in CD8:Treg ratio, which was transient but increased again after the addition of checkpoint blockade. The presence of CD8 T cell infiltrate on day 8 and its marked reduction on day 35 show that initiation of immunotherapy should be applied early in the course of targeted therapy to take advantage of the dense T cell infiltrate early after targeted therapy initiation. This result is limited to a single patient, but has been replicated in the subcutaneous implantable tumor model generated from a well-established murine model of BRAF mutant melanoma^47^.

Whether the addition of MEK inhibition combined with immune checkpoint blockade (MEK inhibitors) suppresses T cell function *in vitro* remains debatable^34^. Studies are currently being conducted to clarify whether this condition will affect potential synergy *in vivo*. However, existing data suggest that the addition of MEK inhibitors to targeted and immunotherapy combinations may be associated with increased toxicity, given that in a recent study, several patients who underwent dabrafenib, trametinib, and ipilimumab treatment developed AEs related to colonic perforation^50^. This result, which was unexpected and found in a limited number of patients, highlights the need to further understand the immunomodulatory effects of trametinib.

In summary, metastatic melanoma treatment may have undergone much development, but this progress has resulted in the complexity of managing melanoma patients. The appropriate timing and sequence with molecularly targeted therapy and immunotherapy remains controversial, and synergy is suggested to exist between the two approaches. However, this synergy is tempered by a potential increase in toxicity. Further studies should be performed to increase the understanding of the responses to these types of therapy, and insights gained will help guide optimal management of melanoma patients.
